# Biomineralized Manganese Oxide Nanoparticles Synergistically Relieve Tumor Hypoxia and Activate Immune Response with Radiotherapy in Non-Small Cell Lung Cancer

**DOI:** 10.3390/nano12183138

**Published:** 2022-09-10

**Authors:** Xinyu Liu, Meron Tsegay Kifle, Hongxin Xie, Liexi Xu, Maoling Luo, Yangyi Li, Zhengrong Huang, Yan Gong, Yuzhou Wu, Conghua Xie

**Affiliations:** 1 Department of Radiation and Medical Oncology, Zhongnan Hospital of Wuhan University, Wuhan 430071, China; 2 Hubei Key Laboratory of Bioinorganic Chemistry and Materia Medica, Hubei Engineering Research Center for Biomaterials and Medical Protective Materials, School of Chemistry and Chemical Engineering, Huazhong University of Science and Technology, Wuhan 430074, China; 3 Department of Biological Repositories, Zhongnan Hospital of Wuhan University, Wuhan 430071, China; 4 Tumor Precision Diagnosis and Treatment Technology and Translational Medicine, Hubei Engineering Research Center, Zhongnan Hospital of Wuhan University, Wuhan 430071, China; 5 Hubei Key Laboratory of Tumor Biological Behaviors, Zhongnan Hospital of Wuhan University, Wuhan 430071, China; 6 Hubei Cancer Clinical Study Center, Zhongnan Hospital of Wuhan University, Wuhan 430071, China; 7 Wuhan Research Center for Infectious Diseases and Cancer, Chinese Academy of Medical Sciences, Wuhan 430071, China

**Keywords:** NSCLC, radiotherapy, nanoparticles, cGAS-STING, radioimmune responses

## Abstract

Radiotherapy (RT) is currently considered as an essential treatment for non-small cell lung cancer (NSCLC); it can induce cell death directly and indirectly via promoting systemic immune responses. However, there still exist obstacles that affect the efficacy of RT such as tumor hypoxia and immunosuppressive tumor microenvironment (TME). Herein, we report that the biomineralized manganese oxide nanoparticles (Bio-MnO_2_ NPs) prepared by mild enzymatic reaction could be a promising candidate to synergistically enhance RT and RT-induced immune responses by relieving tumor hypoxia and activating cGAS-STING pathway. Bio-MnO_2_ NPs could convert endogenic H_2_O_2_ to O_2_ and catalyze the generation of reactive oxygen species so as to sensitize the radiosensitivity of NSCLC cells. Meanwhile, the release of Mn^2+^ into the TME significantly enhanced the cGAS-STING activity to activate radio-immune responses, boosting immunogenic cell death and increasing cytotoxic T cell infiltration. Collectively, this work presents the great promise of TME reversal with Bio-MnO_2_ NPs to collaborate RT-induced antitumor immune responses in NSCLC.

## 1. Introduction

Lung cancer ranks first in the cause of cancer death in countries around the world, with an estimated almost 20% by the end of 2020 [1]. According to histopathologic features, it is divided into small cell and non-small cell lung cancer (NSCLC), with the latter accounting for 80–85% lung cancer cases [2,3]. Despite the remarkable progress that has been achieved on diagnosis and treatment in the last decades, most NSCLC patients are diagnosed at an advanced stage, leading to the suboptimal prognosis. Given that the overall cure rate for NSCLC remains low and the 5-year survival rate is not ideal, it remains to be expected whether more effective treatments can be found [4].

Radiotherapy (RT) is currently a main treatment for NSCLC, approximately half of NSCLC patients receiving RT at least once [5,6]. The basic principle of RT involves radiation-induced damage to biomolecules such as cellular proteins, lipids and especially DNA as well as the generation of reactive oxide species (ROS) due to radiolysis [7,8]. In addition, RT facilitates tumor-associated immune responses. The cyclic GMP-AMP (cGAMP) synthase (cGAS)/stimulator of the interferon genes (STING) pathway has been identified as a role worth studying for exerting antitumor immune responses. It can associate RT-induced DNA damage with the infiltration and activation of cytotoxic T lymphocytes (CTLs) [9,10]. Following RT, increased amounts of cytosolic double-stranded DNA (dsDNA) are detected by cGAS, then cGAMP is produced from ATP and GTP. Upon cGAMP binding, STING is activated and phosphorylates TANK binding kinase (TBK) 1, and then interferon (IFN) regulatory factor (IRF) 3, leading to type I IFN upregulation and induced immune responses [11,12]. Moreover, RT was reported to induce immunogenic cell death (ICD), which is defined as the immunogenic part in all forms of cell death [13]. ICD releases danger associated molecular patterns and tumor-associated antigens to promote maturation of dendritic cells and CTLs infiltration, resulting in attacks to tumor cells [14,15].

However, there still exist obstacles that affect the efficacy of RT. On the one hand, tumor hypoxia, primarily as a result of vascular anomalies, causes more tumor resistance to ionizing radiation than tumor normoxia, since oxygen promotes the formation of DSB [16,17,18,19]. On the other hand, the elevated glutathione (GSH) level is a common feature for cancer cells by contrast with normal cells. The synthesis of GSH is manipulated by cancer cells to prevent the damages of ROS, thereby decreasing DNA damage attributed to oxidative stress and leading to an immunosuppressive tumor microenvironment (TME) [20,21,22]. Therefore, there is an urgent demand to develop low-toxic and highly efficient radio-collaborators that are capable of alleviating tumor hypoxia while reversing immunosuppressive TME.

For the past few years, the rapid development of nanomaterials has provided a wide, developing prospect for sensitizing tumor RT. The ability of nanoparticles to act as radiosensitizers is commonly explained via the enhancement of energy deposition, which is attributed to the increased absorption of X-rays associated with the emission of secondary electrons and fluorescence photons [23,24]. Therefore, high-Z nanomaterials, such as gold nanoparticles (GNPs), have been widely studied for RT [25]. Recently, the applications of manganese dioxide (MnO_2_)-based nano-systems, with outstanding biosecurity and the distinctive physicochemical property, have attracted increasing attention in tumor therapy [26,27]. They were reported to reduce GSH concentration, clear overproduced hydrogen peroxide (H_2_O_2_), induce ROS generation, neutralize internal acidity and generate oxygen to relieve hypoxia in TME [28,29]. In addition, MnO_2_ nano-systems did not present long-term toxicity as they can be broken down into harmless water-soluble Mn^2+^ ions and excreted fast by kidneys [30,31]. Moreover, Mn^2+^ ions were reported to augment the sensitivity of dsDNA to activate the cGAS-STING pathway [32,33], reverse the limitation of TME on the killing function of CTLs and inhibit the immunosuppressive effects [34,35,36]. Consequently, the combination of RT and MnO_2_ nano-systems has great potential to explore for researchers. Thereby, we propose the MnO_2_ as a promising candidate to synergistically reverse tumor hypoxia and promote RT-induced immune responses.

MnO_2_ nanoparticles (NPs) are normally produced by reduction of manganese permanganate, which is often unstable in aqueous solutions and requests surface passivation by polymers or proteins [37,38]. Recently, we reported a facile method to produce MnO_2_ NPs by a biomineralization process catalyzed via a multicopper oxidase called MnxEFG [39]. The MnxEFG complex was identified from Bacillus sp. PL-12 and recombinantly expressed by E. Coli [40,41]. They catalyzed the oxidation of Mn^2+^ to form biomineralized manganese oxide nanoparticles (Bio-MnO_2_ NPs) in a mild condition. The resulted NPs were well dispersed without any further surface modification, since the surface was already passivated by proteins. These NPs had excellent photothermal effects and facilitated MRI imaging of TME due to the pH and GSH responsive release of Mn^2+^ [39].

Herein, we investigated the potential of Bio-MnO_2_ NPs to enhance RT effects and RT-induced immune responses. The efficacy of Bio-MnO_2_ NPs in generating O_2_ from H_2_O_2_ and relieving tumor hypoxia was evaluated both in vitro and in vivo. The synergistic effects of Bio-MnO_2_ NPs and RT in NSCLC cells were also evaluated, and the underlying mechanism was investigated. The results indicated that the Bio-MnO_2_ NPs not only relieved tumor hypoxia so as to augment the efficacy of RT, but also synergistically enhanced the cGAS-STING activity to activate radioimmune responses, boosting ICD while increasing CTLs infiltration. Taking the excellent biocompatibility and facile mild synthesis protocol into account, the strategy could be highly promising to enhance RT effects, and indicates a novel strategy to induce immune responses and achieve tumor-specific therapy.

## 2. Materials and Methods

### 2.1. Bio-MnO_2_ NPs Preparation

The MnxEFG complex was expressed and purified according to previous reports and the LPS and other bacterial related components were removed during MnxEFG purification. To obtain Bio-MnO_2_ NPs, a purified MnxEFG complex (2 mg) was added to 200 mL of biomineralization buffer (HEPES, 10 mM, pH = 7.8; NaCl, 50 mM, followed by the addition of 100 μM MnCl_2_) and vortexed and was kept still for 30 min at 30 °C. The obtained MnO_2_ was centrifuged at 8000 rpm for 10 min using an Amicon Ultra 20 mL centrifugal filter (MWCO = 30 kDa), washed with deionized water twice, before collecting it into centrifugation tubes. The water dispersed particles were spined at 15,000 rpm and washed three times using deionized water to remove the unbound Mnx protein. Finally, Bio-MnO_2_ NPs were dispersed in water for further use via mild ultrasonic treatment. The Cy3 labelled Bio-MnO_2_ NPs were obtained by the same method, only replacing MnxEFG with Cy3-MnxEFG.

### 2.2. Characterization

The morphologies of Bio-MnO_2_ NPs were investigated through transmission electron microscopy (TEM) HT 7700. Dynamic light scattering (DLS) were carried out by Malven Zetasizer. X-ray diffraction (XRD) analyses were performed on SmartLab-SE diffractometer. The Mn concentration was obtained via ICP-OES.

### 2.3. Detection of GSH

To confirm the degradability of Bio-MnO_2_ NPs upon encountering intracellular GSH, Bio-MnO_2_ NPs were dispersed in water and incubated with different concentrations of GSH (0 mM, 0.02 mM, 0.2 mM, 2 mM) for 10 min at 37 °C. The change in absorbance intensity of UV-vis-NIR spectra was recorded on Lambda 750S spectrometer (PerkinElmer, Shelton, CT, USA).

### 2.4. Detection of Oxygen Production

The dissolved oxygen meter (oxygen probe Leici JPSJ-605F, Shanghai, China) was used to examine the oxygen generation ability of Bio-MnO_2_ NPs. To mimic the oxygen production in the tumor microenvironment, Bio-MnO_2_ NPs were dispersed in 10 mL DMEM containing 10% FBS, the dissolved oxygen level was measured at a range of concentrations of Bio-MnO_2_ NPs (0, 25, 50, 100 μg/mL) and Chem-MnO_2_ (25 μg/mL) with H_2_O_2_ solution (2 mM) and at various H_2_O_2_ concentrations (0, 0.25, 0.5, 1 mM) with Bio-MnO_2_ NPs (25 μg/mL).

### 2.5. Cell Culture

The human lung adenocarcinoma cell lines (A549, PC9), human lung squamous carcinoma cell line H520 and the Lewis lung carcinoma (LLC) cells were purchased from the Type Culture Collection (Chinese Academy of Sciences, Shanghai, China). A549, PC9 and H520 were cultured in RPMI-1640 medium, LLC in DMEM medium containing 10% fetal bovine serum (FBS) and 1% penicillin/streptomycin in humidified incubator (37 °C, 5% CO_2_).

### 2.6. Cellular Uptake

NSCLC cells were seeded in confocal dishes at a density of 4 × 10^4^ cells per dish, after 24 h the medium was replaced with 300 µL full media containing the Cy3 labelled Bio-MnO_2_ NPs (25 μg/mL). The cells were further incubated for different time intervals (1, 2, 4 h). Then, the cells were washed two times with phosphate buffered saline (PBS) before adding Hoechst 33342 (0.4 mg/mL) containing PBS. After 10 min, the cells were washed with PBS and covered with 200 μL of full media and the fluorescence imaging was conducted by confocal microscopy.

### 2.7. Measurement of Intracellular H_2_O_2_

NSCLC cells were plated into 6-well plates and incubated overnight. After added with Bio-MnO_2_ NPs for 4 h, cells were irradiated (8 Gy) using the small animal radiation research platform (SARRP, PXI X-RAD 225Cx, North Branford, CT, USA) with X-ray energy of 225 kV, current beam of 13 mA, dose rate of 1.3 Gy/min and source-surface distance (SSD) of 60 cm and co-cultured for another 24 h. After mixed with H_2_O_2_ lysis solution, the blends were centrifuged to take the supernatant, leading to the harvest of cell samples with different treatments. Then, after adding the sample and H_2_O_2_ detection reagent to each well, they were placed at room temperature for 30 min to detect the absorbance at 560 nm with the microplate reader. Finally, the concentration of H_2_O_2_ were calculated via the standard curve which were drawn in advance.

### 2.8. ROS Detection In Vitro

For flow cytometry assays, NSCLC cells in the 6-well plates were added with Bio-MnO_2_ NPs for 4 h before exposed to 8 Gy. After 24 h, cells were harvested and washed with PBS, while working solution containing DCFH-DA probe (1:1000) were prepared with serum-free medium for resuspending cells. The blends were kept in the dark at 37 °C for 20 min, and were mixed once every 5 min to allow a full reaction. After incubation, the samples were centrifuged and washed 3 times with serum-free medium to avoid specific background fluorescent signals. Subsequently, the samples were resuspended by serum-free medium and fluorescence signals were collected via the flow cytometer.

For fluorescence image acquisition, the treated cells were washed with PBS for twice and were stained with working solution prepared in advance for 20 min at 37 °C, then washed 3 times with serum-free medium and observed using a fluorescence microscope.

### 2.9. Cytotoxicity In Vitro

Cytotoxicity based on the Bio-MnO_2_ NPs or the collaborative RT studies were estimated through the cell counting kit-8 (CCK-8) assay. NSCLC Cells (5000 cells per well) were plated into 96-well plates. After cultured for 24 h, the cells were incubated with various concentrations of Bio-MnO_2_ NPs (0, 5, 10, 25, 50, 100, 150, 200 μg/mL) for another 4 h and received X-rays (8 Gy) or not and further incubation for subsequent 24 h. Subsequently, each well was added with 100 μL precast mixed solution containing 10 μL CCK-8, the optical density value of the cells at 450 nm was detected via the microplate reader. 

### 2.10. Cell Apoptosis Assay

After adhesion NSCLC cells were treated with Bio-MnO_2_ NPs and followed by radiation. After 24 h of continuous incubation, cells and their supernatants were centrifuged to collect and washed with PBS. The harvested samples were stained with Annexin V-FITC solution for 15 min and propidium iodide solution for another 5 min in the dark on ice. The cells were analyzed via flow cytometry.

### 2.11. Colony Formation Assay

NSCLC cells were plated into 6-well plates and incubated overnight. After added with Bio-MnO_2_ NPs for 4 h, cells received radiation and co-cultured for another 24 h. The samples of each group were digested and re-inoculated into 6-well plates at a density of 1000 cells/well. After culturing for 14 days, the colonies were fixed with 4% paraformaldehyde and stained with crystal violet. 

### 2.12. RNA Isolation and qRT-PCR

Total RNA was extracted from NSCLC cells employing TRIZOL. After the assessment of the RNA concentration and quality via the Nanodrop spectrophotometer, the RNA was reversely transcribed into cDNA by the Reverse Transcriptase Kit and a reaction system containing ChamQTM SYBR^®^ qPCR Master Mix, primers (See Table S1), cDNA and ddH_2_O was configured to perform qRT-PCR. The relative expression of mRNA was assessed via the 2^−ΔΔCt^ method. 

### 2.13. Immunoblotting

For the extraction of proteins, NSCLC cells were fully lysed on ice using RIPA buffer including 1% protease inhibitor and phosphatase inhibitor for half an hour, after collected by centrifugation, the supernatants were mixed with loading buffer to obtain protein samples. Proteins were electrophoresed using SDS-PAGE and transferred to PVDF membranes. After blocked with 5% skimmed milk, the membranes were incubated with primary antibody overnight at 4 °C with gentle shaking. After being washed by TBST, the membrane was incubated with the corresponding secondary antibody for 1 h. After washed by TBST again, bands were captured using a chemiluminescence (ECL) detection system.

### 2.14. Immunofluorescence

NSCLC cells adhered on glass slides were washed with PBS and fixed with 4% paraformaldehyde for 30 min, permeabilized with 0.5% Triton X-100 for 20 min and then blocked with 5% bovine serum albumin for 1 h. After overnight incubated with the primary antibody at 4 °C and washed by PBST, the cells were stained with the secondary antibody for 1 h. Representative images were collected employing the fluorescence microscope and the laser confocal microscope.

### 2.15. Comet Assay

The treated NSCLC cells were centrifuged to collect pellet. After being washed by ice-cold PBS, the samples were resuspended with molten agarose before application to the OxiSelectTM Comet Slide. The embedded cells were treated with the pre-chilled lysis buffer for 1 h and alkaline solution for 30 min at 4 °C in the dark. Then the samples were electrophoresed in electrophoretic buffer. Following electrophoresis, the samples were coexistent with cold 70% Ethanol and once the slides were completely dry, the agaroses were stained with DNA dye. Cells were visualized via fluorescent microscope and analyzed by CASPlab.

### 2.16. Enzyme-Linked Immunosorbent Assay (ELISA)

The NSCLC cell supernatants were collected after treatment. The samples were detected using the ELISA kit, and then after measuring the absorbance (OD values), the levels of cytokines were calculated according to the standard curve.

### 2.17. Mice Model

C57BL/6 mice, 5 weeks of age, female, were purchased from the Jiaxing Wanqian Biology Technology. About 1 × 10^7^ LLC cells suspended in serum-free medium for each mouse were implanted subcutaneously into the right hip. Tumor volume was measured once a day according to the formula: Volume = (length × width^2^)/2. The animal experiments were accomplished according to the guidelines of the Institutional Animal Care and Use Ethical Committee at center of Wuhan University Animal Experiment. 

### 2.18. In Vivo Safety Analysis

When the mice reached the welfare end point, the body weight was measured and recorded. For the detection of the blood routine, the blood of mice was collected intravenously in an anticoagulation tube filled with heparin, and the data were obtained by the superior detection. For the detection of the liver and kidney function, the supernatants of the blood samples were analyzed via ELISA kits. The organs (heart, liver, spleen, lungs and kidneys) from mice were dissected and encapsulated in paraffin and stained by H&E.

### 2.19. In Vivo Antitumor Efficacy

When the tumor volume approached approximately 600 mm^3^, the LLC bearing mice were randomly distributed to four groups: (1) negative control (NC); (2) Bio-MnO_2_ NPs; (3) IR (8 Gy × 3); and (4) IR + Bio-MnO_2_ NPs. The mice accepted intratumor injection of Bio-MnO_2_ NPs (2 mg/kg) or PBS, in addition, 4 h after injection the mice in groups containing IR accepted the X-ray radiation of 8 Gy using the small animal radiation research platform (PXI X-RAD 225Cx) with the SARRP via a single collimated field prescribed to midplane. The beam was 225 kV and 13 mA X-ray energy at 2.7 Gy/min. The SSD was 30 cm and the radiation field was 2 cm × 2 cm. The steps were as follows: the mice accepted intraperitoneal injection of the anesthetic prepared with sodium pentobarbital powder (40 mg/kg), then the mice were fixed on the partition, the irradiation time were calculated according to the tumor depth and the height of the light-limiting barrel and the position was adjusted under CT guidance. For the next two days, the administration and IR in vivo were repeated and the mice were treated three times altogether. Tumor volume was measured once a day until the mice reached the welfare end point.

The IVIS Lumina system with luciferase-mediated bioluminescence was used to measure the tumor volume in vivo. Tumors from all mice were dissected for further measuring and photographing. Tumor tissues were encapsulated in paraffin and stained by H&E, Ki-67, TUNEL, dsDNA, CD3 and CD8.

The flow cytometry assay was employed to estimate the immune cells. Spleen tissues were pulverized, added with erythrocyte lysate and filtered to obtain single-cell suspension. Meanwhile, tumor tissues were minced and digested with collagenase IV (Sigma-Aldrich, St. Louis, MO, USA). After blocking in serum, cells were stained with Fixable Viability Stain to separate viable cells, and then they were stained with fluorescein-conjugated antibodies (See Table S2) against CD45, CD3, CD8 and CD4 for 30 min at 4 °C. Fluorescence signals were collected via the flow cytometer and analyzed via FlowJo software. The sera of the mice were also harvested before execution to evaluate the level of cytokine via ELISA assay.

### 2.20. Statistical Analysis

Statistical analysis was studied via GraphPad Prism 8 (founded by Dr. Harvey Motulsky, San Diego, CA, USA). Student’s *t*-tests were employed to determine the differences between 2 groups. All data results were presented as mean ± standard deviations (SD). *p* < 0.05 was considered as statistically significant.

## 3. Results and Discussion

### 3.1. Characterization of Bio-MnO_2_ NPs

**Figure 1:**
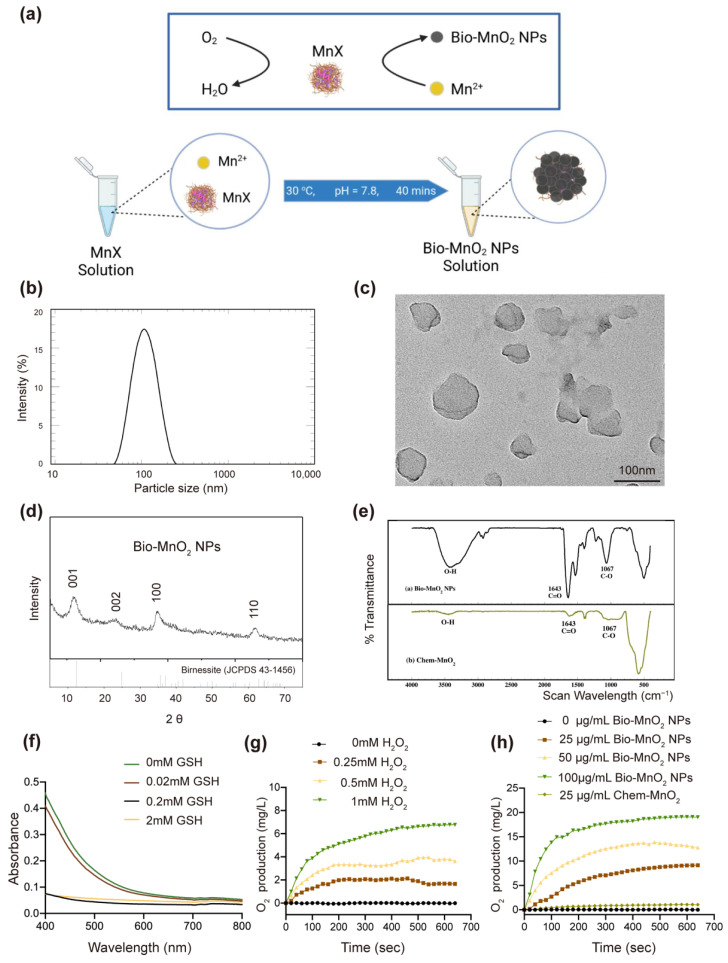
Characterization of biomineralized manganese oxide nanoparticles (Bio-MnO_2_ NPs). (**a**) Fabrication of Bio-MnO_2_ NPs via MnxEFG mediated biomineralization. (**b**) dynamic light scattering (DLS) measurement of size of Bio-MnO_2_ NPs. (**c**) transmission electron microscopy (TEM) of NPs formed by biomineralization of MnxEFG. (**d**) X-ray diffraction (XRD) patterns for Bio-MnO_2_ NPs prepared via MnxEFG catalysis. (**e**) FT-IR spectra of Bio-MnO_2_ and Chem-MnO_2_. (**f**) Reaction between GSH (0, 0.02, 0.2, 2 mM) and Bio-MnO_2_ NPs. (**g**) O_2_ generation at various H_2_O_2_ concentrations (0, 0.25, 0.5, 1 mM) with Bio-MnO_2_ NPs (25 μg/mL). (**h**) O_2_ generation in different concentrations of Bio-MnO_2_ NPs (0, 25, 50, 100 μg/mL) and Chem-MnO_2_ (25 μg/mL) with H_2_O_2_ solution (2 mM).

Via a biomineralization process the Bio-MnO_2_ NPs were obtained (Figure 1a). The recombinant multicopper oxidase MnxEFG was expressed and purified from E. coli. MnxEFG was added to the biomineralization buffer (HEPES, 10 mM, pH = 7.8; NaCl, 50 mM; MnCl_2_, 100 μM). The solution was incubated at 30 °C quiescently to allow homogeneous production of Bio-MnO_2_ NPs. According to our previous study [39], the average size of NPs was controlled from 60 to 200 nm depending on the incubation time from 30 to 60 min. In order to facilitate sufficient passive targeting to solid tumors via EPR effect, while allowing a more complete biomineralization, the biomineralization process was halted after 40 min. The average hydrodynamic diameter of the NPs was 101 nm as measured by DLS with a polydispersity (PdI) value of 0.122 (Figure 1b), indicating the size distribution of these nanoparticles could be ideal for tumor penetration and well acceptable for clinical applications due to its extravasation through vascular fenestrations of tumors and escape from filtration by the liver and spleen [42]. Furthermore, the morphology of the NPs was analyzed using TEM (Figure 1c). The XRD patterns of the Bio-MnO_2_ NPs showed four major peaks corresponding to hexagonal birnessite (JCPDS 43-1456) (Figure 1d). This pattern matched the hexagonal and turbostratic birnessite, and the splitting for picks at (100) and (110) and the d-values (1.73) between these 2 planes were absent for biogenic hexagonal and turbostratic birnessite [39]. The FT-IR (Figure 1e) showed the high content of the organic component due to the encapsulation of the enzymes during biomineralization process, which might explain its excellent dispersion in water as opposed to Chem-MnO_2_.

Moreover, while maintaining their integrity in the concentration range of GSH mimicking the extracellular environment [43,44,45], these NPs could degrade within few minutes upon encountering GSH in the intracellular concentration range [43,44,45] (Figure 1f). This process would be beneficial to reverse immunosuppressive TME by neutralizing inner tumor acidity and consuming GSH. Moreover, the MnO_2_ NPs were desired to relieve tumor hypoxia by oxidizing overproduced H_2_O_2_ in TME to O_2_. Therefore, the oxygen production efficiency was investigated with a series of concentrations of H_2_O_2_ and Bio-MnO_2_ NPs. With the increasing concentrations of H_2_O_2_, more O_2_ was produced by Bio-MnO_2_ NPs in a short time (equilibration reached within 10 min) (Figure 1g). With the same concentration of H_2_O_2_, the higher concentration of Bio-MnO_2_ NPs resulted in significantly elevated O_2_ production (Figure 1h). As a comparison, the same concentration of Chem-MnO_2_ only produced <10% oxygen, which clearly indicated the high oxidation efficiency of Bio-MnO_2_ NPs. This phenomenon was possibly benefit from the superior water dispersity as well as the more Mn vacancies presented in Bio-MnO_2_ NPs that led to faster reaction.

### 3.2. Bio-MnO_2_ NPs Eliminated H_2_O_2_ and Enhanced ROS Production in NSCLC Cells

**Figure 2:**
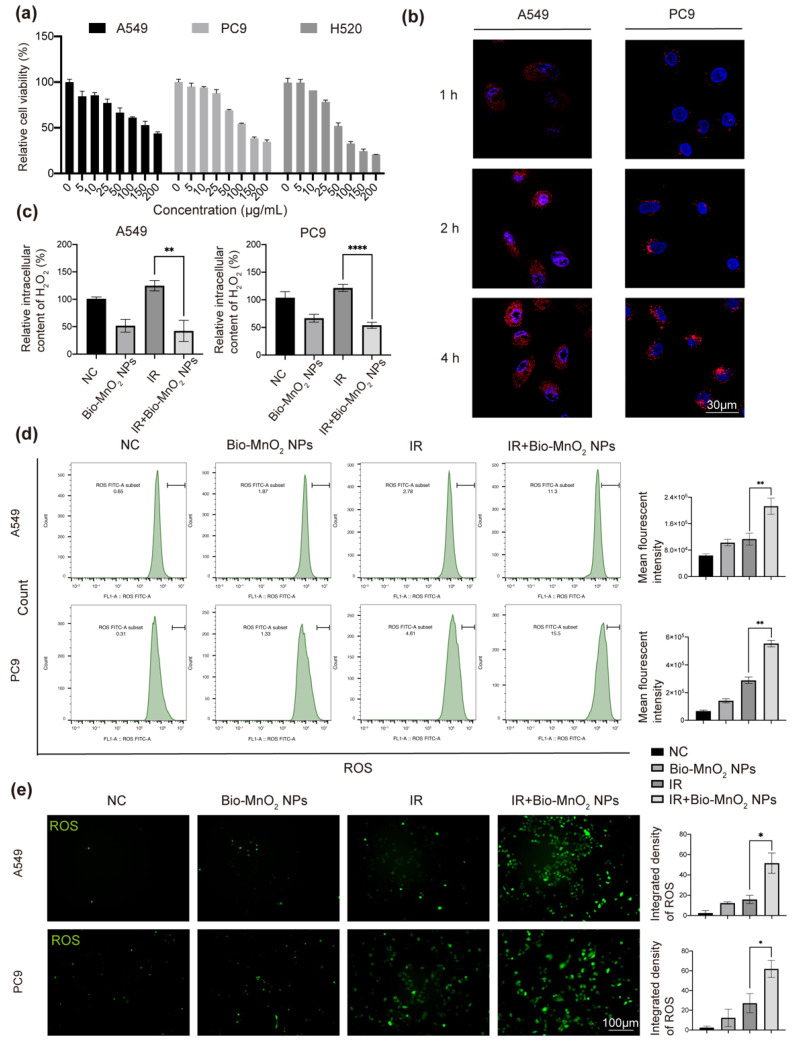
Bio-MnO_2_ NPs eliminated H_2_O_2_ and enhanced reactive oxide species (ROS) production in non-small cell lung cancer (NSCLC) cells. (**a**) Cell viabilities of A549, PC9 and H520 cells treated with different concentrations of Bio-MnO_2_ NPs for 24 h. (**b**) Uptake of Cy3 labelled Bio-MnO_2_ NPs at different time intervals (1 h, 2 h, 4 h). Scale bar, 30 μm. (**c**) Relative intracellular H_2_O_2_ content after incubation with Bio-MnO_2_ NPs (25 μg/mL) for 4 h with or without irradiation (IR) (8 Gy). (**d**) Flow cytometry of ROS probe in NSCLC cells after different treatments as above. (**e**) Representative DCF staining images of NSCLC cells. Scale bar, 100 μm. The combination of Bio-MnO_2_ NPs and IR significantly enhanced ROS production than individual treatment. *, *p* < 0.05; **, *p* < 0.01; ****, *p* < 0.0001.

Encouraged by the results in solution, we further tested the oxidation efficiency of Bio-MnO_2_ NPs in NSCLC cells. First, we co-incubated NSCLC cells (A549, PC9 and H520) with different concentrations of Bio-MnO_2_ NPs to evaluate the biocompatibility. Up to 25 μg/mL of Bio-MnO_2_ NPs were used, which did not lead to significant cytotoxicity (Figure 2a). The observed slight toxicity was likely due to the chemo-dynamic effects of released Mn^2+^ as reported by the other literature [46,47,48,49,50]. By labeling the MnxEFG enzyme with Cy3, fluorescent Bio-MnO_2_ NPs were produced and used to confirm the clear uptake of Bio-MnO_2_ NPs into A549 and PC9 cells according to confocal imaging at different time intervals (Figure 2b and Figure S1). After 4 h, we observed the highest fluorescent intensity. Therefore, 4 h was used for the subsequent studies. Thereafter, the intracellular concentration of H_2_O_2_ was evaluated by absorbance measurement (Figure 2c). The addition of 25 μg/mL Bio-MnO_2_ NPs led to approximately 30–50% decreasing of the H_2_O_2_ content comparing to untreated cells. Moreover, IR induced H_2_O_2_ could also be suppressed to more than 50% by Bio-MnO_2_ NPs. These results indicated that the Bio-MnO_2_ NPs could efficiently consume H_2_O_2_ as expected.

The oxygen production from H_2_O_2_ oxidation is preferred to promote ROS generation. The excess of ROS can produce oxidative stress resulting in fatal harm to organelles and DNA, also enhance the effect of RT [51]. Therefore, the DCFH-DA probe was employed for the detection of ROS production. Via both flow cytometry (Figure 2d and Figure S2a) and fluorescent imaging analysis (Figure 2e and Figure S2b) the green fluorescent intensity was found only slightly increased when treated by either Bio-MnO_2_ NPs or IR, indicating insignificant production of ROS by single treatment. Interestingly, combining Bio-MnO_2_ NPs together with IR led to significantly enhanced ROS production, which indicated the synergistic effects of the 2 treatments. The dramatic enhancement corresponded well with our hypothesis that the increased oxygen production from Bio-MnO_2_ NPs was beneficial to generate more ROS from IR. Moreover, some of the literature also proposed that the photon and water might produce some of excited species which could diffuse to the water–NPs interface where their excitation energy could produce ROS [52]. In addition, we speculated the effect observed might relate to X-ray tube voltage, dose rate and particle size, as observed by others [53]. Nevertheless, it provides us with a new construct to improve the effect of RT by determining the optimal radiation conditions such as size of nanomaterials and dose rate.

### 3.3. Bio-MnO_2_ NPs Enhanced Radiosensitivity of NSCLC Cells In Vitro

**Figure 3:**
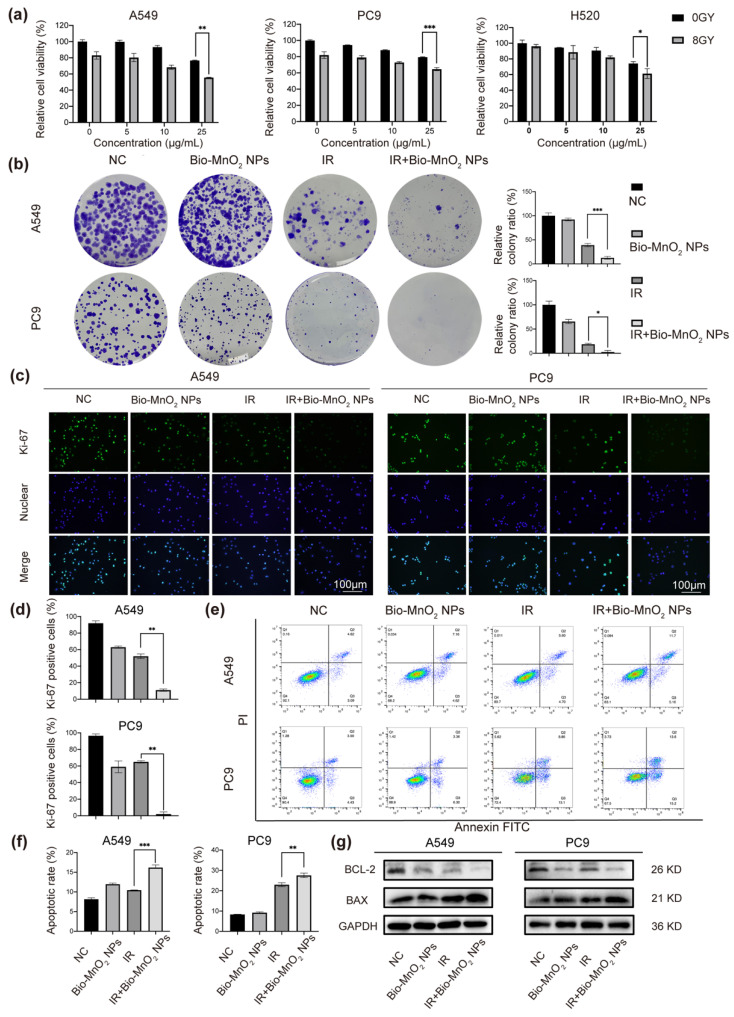
Bio-MnO_2_ NPs enhanced radiotherapy (RT) in vitro. (**a**) Cell viabilities of A549, PC9 and H520 cells incubated with Bio-MnO_2_ NPs at various concentrations with or without X-ray radiation. (**b**) Colony formation assay of NSCLC cells after different treatments and statistical graphs of colony formation assay. (**c**,**d**) Representative images and statistical graphs of Ki-67 immunofluorescence. Scale bar, 100 μm. (**e**,**f**) Cell apoptosis assay via flow cytometry. (**g**) Immunoblotting of cell apoptosis related proteins. *, *p* < 0.05; **, *p* < 0.01; ***, *p* < 0.001.

To evaluate the RT sensitizing ability of Bio-MnO_2_ NPs, we chose A549, PC9 and H520 cells to incubate with various concentrations of Bio-MnO_2_ NPs, and then exposed the cells to 8 Gy of radiation. Compared to the cells only received IR, pre-incubation with Bio-MnO_2_ NPs resulted in higher cell mortality with clear concentration dependency (Figure 3a), suggesting that Bio-MnO_2_ NPs could exacerbate tumor cell damage acting as radiosensitizers. The IR dose of 8 Gy together with 25 μg/mL Bio-MnO_2_ NPs showed most significant synergistic effect, which was therefore used for the subsequent in vitro studies.

The radiosensitizing ability of Bio-MnO_2_ NPs was also verified by colony formation assay. Compared with cells only exposed to IR, a remarkable reduction in the number of colonies was observed in NSCLC cells co-incubated with Bio-MnO_2_ NPs before IR (Figure 3b), again indicating the Bio-MnO_2_ NPs could enhance the cell damage induced by IR. We also found that the combinatorial treatment with both IR and Bio-MnO_2_ NPs led to dramatic decreased Ki-67 expression in both A549 and PC9 cells [54,55], which was clearly more significant than the simple accumulative effect of each single treatment (Figure 3c,d). These results again supported the beneficial sensitizing effect of Bio-MnO_2_ NPs for RT.

Radiation-induced apoptosis as the major cell death mechanism involved in cancer RT was also investigated. Indeed, the Bio-MnO_2_ NPs and IR combined treatment resulted in the highest ratio of apoptosis compared to either single treated group (Figure 3e,f). Immunoblotting also revealed the higher levels of BAX protein and lower levels of BCL-2 protein (Figure 3g), the family of which is considered as major regulators of apoptosis [56,57]. The BAX/BCL-2 ratio was also found augmented, indicating increasing activation of apoptotic pathways.

Overall, the above results demonstrated that Bio-MnO_2_ NPs exerted a synergistic impact in combination with RT.

### 3.4. Bio-MnO_2_ NPs plus Radiation Increased DNA Damage in NSCLC Cells

**Figure 4:**
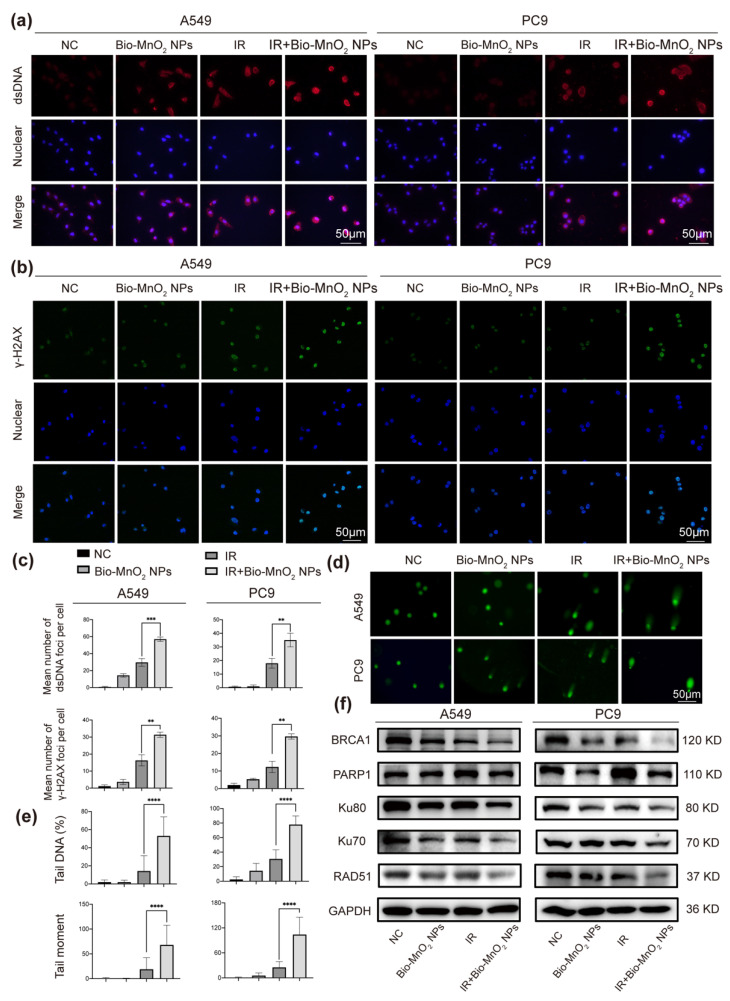
Bio-MnO_2_ NPs plus radiation increased DNA damage in NSCLC cells. (**a**) Representative images of double-stranded DNA (dsDNA) immunofluorescent staining (red) after different treatments. Scale bar, 50 μm. (**b**) Representative images of γ-H2AX immunofluorescence (green) after different treatments. Scale bar, 50 μm. (**c**) Statistical graphs of dsDNA and γ-H2AX immunofluorescence. (**d**) Representative images of comet assay after different treatments in NSCLC cells. (**e**) Statistical graphs of comet assay. (**f**) Immunoblotting of DNA damage and repair related proteins. **, *p* < 0.01; ***, *p* < 0.001. ****, *p* < 0.0001.

RT leads to excessive DNA damage, containing oxidative base damage, single-strand breaks (SSBs) and DSBs, which can eradicate tumor cells [58]. Therefore, the effect of different treatments on DNA damage was further evaluated. First, the immunofluorescence of the dsDNA in the cytoplasm was used to reflect DNA damage of tumor cells. The images suggested that the group combined Bio-MnO_2_ NPs and IR led to the most significant DNA damage as shown by the highest fluorescent intensity of dsDNA in cytosol (Figure 4a,c). Moreover, the phosphorylation of H2AX was also evaluated, which is known as a marker for DSBs and has a close correlation with the number of DNA damage [59]. The results manifested that the Bio-MnO_2_ NPs plus IR showed the most effective activation of the γ-H2AX (Figure 4b,c), which again suggested the radio induced DNA damage that enhanced by Bio-MnO_2_ NPs. Furthermore, the significant DNA damage was also confirmed by comet assay, which works with pre-embedded cells in agarose gel. During electrophoresis, the highly damaged nucleus with fragmented DNA will migrate out from the cell showing a long tail, which could reflect to SSBs, DSBs as well as oxidative lesions [60]. The results revealed that the combinatorial treatment induced remarkable DNA damage in NSCLC cells as evidenced by an increased ratio of tail DNA as well as longer tail moment (Figure 4d,e). Notably, in all three experiments, Bio-MnO_2_ NPs showed almost negligible DNA damage, which suggested the Bio-MnO_2_ NPs did not directly cause DNA damage, but function as a sensitizer to enhance the RT-induced DNA damage. All these results forcefully supported that the Bio-MnO_2_ NPs sensitized the irradiation therefore caused efficient DNA damage to the cells.

Furthermore, we verified the repair of DNA damage. Two distinct pathways are known to eliminate DSBs: non-homologous end-joining (NHEJ) and homologous recombination repair (HRR) [58]. As revealed, the Bio-MnO_2_ NPs and radiation combined treatment significantly downregulated not only the essential members in NHEJ: Ku80, Ku70 and PARP1, but also the important ones in HRR: BRCA1, Rad51 (Figure 4f). Therefore, it is highly attractive that Bio-MnO_2_ NPs increased DNA damage and inhibited DNA damage repair when associated with radiation in NSCLC cells.

### 3.5. Bio-MnO_2_ NPs plus Radiation Enhanced the Activation of cGAS/STING Signaling Pathway

It was proved that cGAS can bind to cytosolic dsDNA and mediate the activation of STING/TBK1/IRF3 pathway leading to the secretion of interferon β (IFN-β) subsequently [61]. Since we observed an accumulation of the cytosolic dsDNA, we validated whether the cGAS/STING signaling pathway was activated.

**Figure 5:**
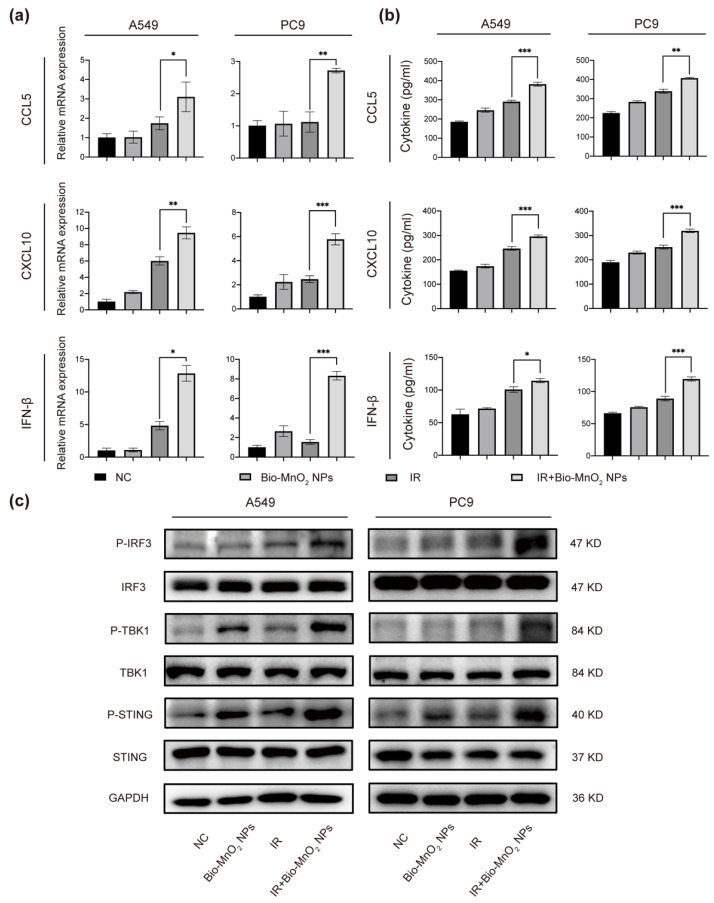
Bio-MnO_2_ NPs plus radiation enhanced the activation of cGAS/STING signaling pathway. (**a**) The mRNA levels of C-C motif chemokine ligand (CCL) 5, CXC motif chemokine ligand (CXCL) 10 and interferon β (IFN-β) after different treatments in A549 and PC9 cells. (**b**) The secretion of CCL5, CXCL10 and IFN-β detected by Enzyme-linked immunosorbent assay (ELISA) after different treatments in A549 and PC9 cells. (**c**) Immunoblotting of the classical cGAS/STING pathway proteins in A549 and PC9 cells. *, *p* < 0.05; **, *p* < 0.01; ***, *p* < 0.001.

We performed qRT-PCR to detect the production of cytokines in cGAS/STING pathway. C-C motif chemokine ligand (CCL) 5, CXC motif chemokine ligand (CXCL) 10 and IFN-β were greatly enhanced in the combined group (Figure 5a). Moreover, ELISA assays indicated that radiation plus Bio-MnO_2_ NPs exhibited a higher quantity of cytokine secretion (Figure 5b). In addition, we evaluated the levels of protein expression in cGAS/STING pathway by immunoblotting. The results suggested that the P-STING, P-TBK1 and P-IRF3 were significantly induced in NSCLC cells upon radiation plus Bio-MnO_2_ NPs compared with single treatments (Figure 5c). To sum up, all these results strongly supported that the Bio-MnO_2_ NPs combined with radiation markedly activated cGAS/STING signaling pathway in NSCLC cells. Since the cGAS-STING pathway play a vital role in regulating immune response produced by type I IFN, it has attracted increasing attention as a promising novel target for cancer immunotherapy. Therefore, the Bio-MnO_2_ NPs could be a possible breakpoint for anti-tumor immunotherapy as an activator of the cGAS-STING pathway, which synergistically enhanced the radiosensitivity of NSCLC cells.

### 3.6. Bio-MnO_2_ NPs plus Radiation Induced ICD

ICD involves exposure of calreticulin (CRT) in cell surface and the release of mediators containing nonhistone chromatin protein high-mobility group box 1 (HMGB1) and adenosine triphosphate (ATP), acts as a prominent pathway to stimulate the immune response to fight cancer [62]. It was recorded that either MnOx or RT can induce ICD, we therefore speculated that the combination of the two could elicit stronger ICD [48,63,64]. 

**Figure 6:**
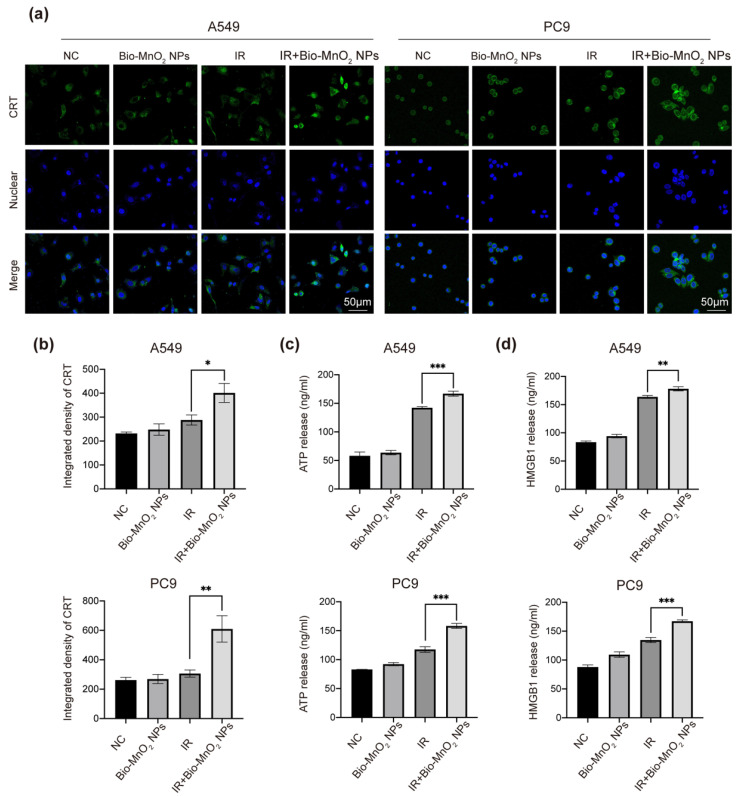
Bio-MnO_2_ NPs plus radiation induced immunogenic cell death (ICD). (**a**,**b**) Representative images and statistical graphs of calreticulin (CRT) immunofluorescence (green) in different groups. Scale bar, 50 μm. (**c**) The secretion of ATP detected by ELISA after different treatments in NSCLC cells. (**d**) The secretion of high-mobility group box 1(HMGB1) detected by ELISA. *, *p* < 0.05; **, *p* < 0.01; ***, *p* < 0.001.

Consequently, we estimated the ability of Bio-MnO_2_ NPs to synergize with RT to trigger ICD via metrics mentioned above. As shown, immunofluorescence revealed that radiation plus Bio-MnO_2_ NPs manifested more CRT exposure than the free radiation treated group (Figure 6a,b). Consistent with the results of the CRT detection, Bio-MnO_2_ NPs plus radiation dramatically increased the release of HMGB1 and ATP according to ELISA assays (Figure 6c,d). The results indicated that combined treatment could synergistically induce ICD in NSCLC cells. 

### 3.7. Bio-MnO_2_ NPs Enhanced RT In Vivo

**Figure 7:**
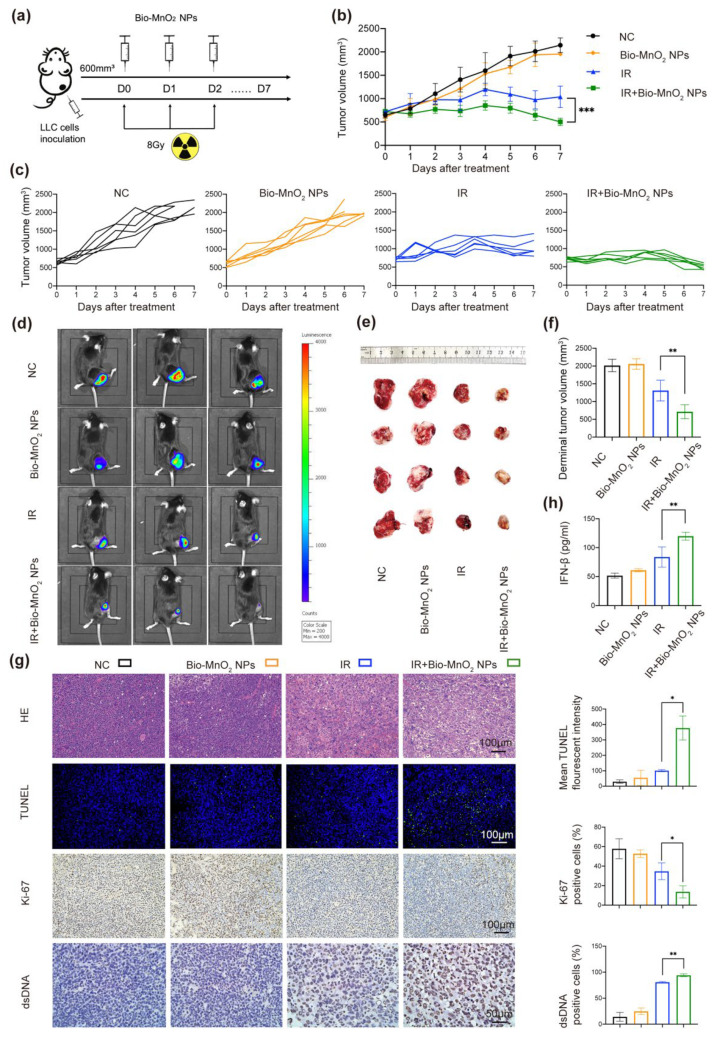
Bio-MnO_2_ NPs enhanced RT in vivo. (**a**) Schematic illustration of the experiment in vivo. (**b**) Growth curve of tumor volume after different treatments: negative control (NC), Bio-MnO_2_ NPs, IR and IR + Bio-MnO_2_ NPs. (**c**) Tumor growth curve of each mouse. (**d**) Representative fluorescence of tumors. (**e**) Representative images of tumors. (**f**) Tumor volumes from each group when the mice reached the welfare end point. (**g**) Representative images and statistical graphs of H&E, Ki-67, TUNEL, dsDNA staining of tumors tissues. (**h**) Cytokine levels of IFN-β in sera after different treatments. *, *p* < 0.05; **, *p* < 0.01; ***, *p* < 0.001.

In order to further evaluate the anti-tumor ability of radiation and Bio-MnO_2_ NPs in vivo, tumor-bearing mice models were built via subcutaneous injection of LLC cells into C57BL/6 mice (Figure 7a), which were randomly distributed to 4 groups: (1) NC; (2) Bio-MnO_2_ NPs; (3) IR; (4) IR + Bio-MnO_2_ NPs.

The Bio-MnO_2_ NPs-mediated radiotherapeutic treatment manifested the most significant effect of inhibiting the rapid tumor growth (Figure 7b). The tumor growth curve of each mouse was exhibited in (Figure 7c and Figure S5). Using the luciferase-mediated bioluminescence system we observed the obvious shrink of tumor area in combined group (Figure 7d), just consistent with the results of representative photos and volumes of tumor tissues (Figure 7e,f).

According to hematoxylin and eosin (H&E) staining, the combined group showed the most severe cell damage (Figure 7g). In addition, we performed TUNEL fluorescent staining to examine the cell apoptosis. As manifested (Figure 7g), there was more green-fluorescence signals in the Bio-MnO_2_ NPs and IR combined group, indicating more apoptosis. The results of Ki-67 staining indicated that Bio-MnO_2_ NPs plus radiation substantially inhibited cell proliferation (Figure 7g). Moreover, the positive rate of dsDNA in tumors was markedly increased after the combined treatment (Figure 7g), and the levels of inflammatory cytokine IFN-β in the serum manifested a high expression in the combined group (Figure 7h).

**Figure 8:**
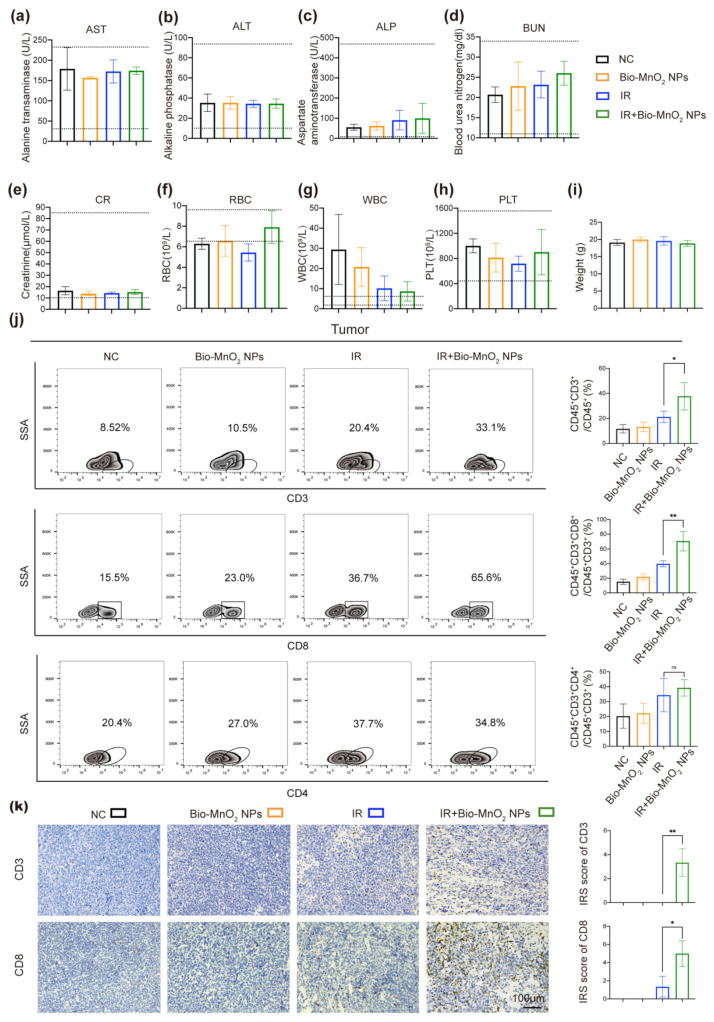
Bio-MnO_2_ NPs plus radiation activated immune responses in vivo. (**a**–**e**) Comparison of liver (ALT, AST, ALP) and kidney function (BUN, CR) in different groups. (**f**–**h**) Comparison of the blood routine (RBC, WBC, PLT) in different groups. (**i**) Body weight of the mice when the mice reached the welfare end point. (**j**) Flow cytometry of CD45^+^CD3^+^, CD45^+^CD3^+^CD8^+^ and CD45^+^CD3^+^CD4^+^ T cells in tumor tissues. (**k**) Representative images and statistical graphs of CD3, CD8 staining in tumors tissues. ns, no significance; *, *p* < 0.05; **, *p* < 0.01.

In addition, we performed a series of experiments to estimate the biological compatibility of Bio-MnO_2_ NPs in vivo. The alanine transaminase (ALT), aspartate aminotransferase (AST) and alkaline phosphatase (ALP) markers were normal, indicating the negligible damage to the hepatic function (Figure 8a–c). The levels of blood urea nitrogen (BUN) and creatinine (CREA) were also within the reference range in all groups, pointing to the acceptable renal functions (Figure 8d,e). The results of the blood routine indicated the normalization of red blood cell (RBC) counts and white blood cell (WBC) counts in the combined group (Figure 8f,g), reflecting the better state of living mice. The inspection results of platelet (PLT) manifested no obvious myelosuppression occurring (Figure 8h). Furthermore, we observed no significant differences in the body weight of mice between the 4 groups (Figure 8i). Moreover, H&E staining were utilized to detect the major organ (heart, liver, spleen, lung and kidney) toxicity. No evident histopathological abnormalities were found (Figure S3), indicating the biosecurity of Bio-MnO_2_ NPs.

### 3.8. Bio-MnO_2_ NPs plus Radiation Activated Immune Responses In Vivo

Given the ability of Bio-MnO_2_ NPs to activate cGAS-STING pathway in vitro that is crucial for immunotherapy, we verified whether Bio-MnO_2_ NPs could also enhance immune responses after RT in vivo. By means of flow cytometry we estimated the ratio of total T cells (CD45^+^CD3^+^) and cytotoxic T cells (CD45^+^CD3^+^CD8^+^) in tumor and spleen tissues. We observed that Bio-MnO_2_ NPs plus radiation significantly augmented both total and cytotoxic T cells in both tumors and spleens (Figure 8j and Figure S4). The results of immunohistochemistry were accordant with flow cytometry, revealing the increased total and cytotoxic T cells in tumor tissues (Figure 8k), the IRS score was utilized to estimate the ratio and intensity of CD3 or CD8 positive cells [65]. However, CD45^+^CD3^+^CD4^+^ T cells were not markedly induced in the combined group (Figure 8j), which perhaps indicating that CD4^+^ T cells might not play a main role in combined treatment-induced immunity. Taken together, Bio-MnO_2_ NPs plus radiation could actuate T cell-related immune responses especially in cytotoxic T cells. 

### 3.9. Cytotoxic T Cells Played an Important Role in Immune Responses Activated by Bio-MnO_2_ NPs Plus Radiation In Vivo

**Figure 9:**
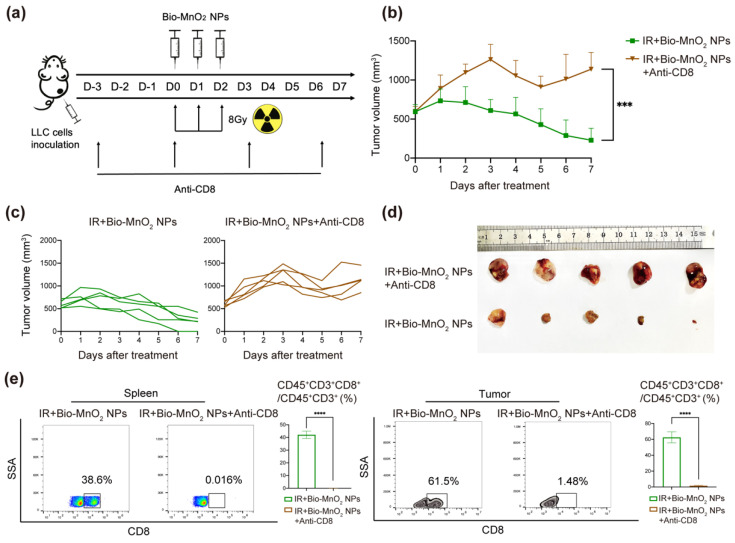
Cytotoxic T cells played an important role in immune responses activated by Bio-MnO_2_ NPs plus radiation in vivo. (**a**) Schematic illustration of the experiment in vivo. (**b**) Growth curve of tumor volume after different treatments: IR + Bio-MnO_2_ NPs, IR + Bio-MnO_2_ NPs + Anti-CD8. (**c**) Tumor growth curve of each mouse. (**d**) Representative images of tumors. (**e**) Flow cytometry analysis of cytotoxic T cells in spleen and tumor tissues. ***, *p* < 0.001; ****, *p* < 0.0001.

To identify the effects of cytotoxic T cells in immune responses activated by Bio-MnO_2_ NPs plus radiation in vivo, the tumor-bearing mice models were established as what mentioned above, and the mice were randomly distributed to 2 groups: (1) IR + Bio-MnO_2_ NPs; (2) IR + Bio-MnO_2_ NPs + Anti-CD8. The mice in the latter group accepted the administration of CD8 antibody (10 mg/kg) via intraperitoneal injection 3 days before the start of treatment and every 3 days thereafter, which was to inhibit the function of cytotoxic T cells (Figure 9a). Administration of the CD8 antibodies impaired the delay of tumor progression in Bio-MnO_2_ NPs-mediated radiotherapeutic treatment (Figure 9b,c), which verified the necessity of cytotoxic T cells. The representative photos of tumor tissues were shown in Figure 9d. Flow cytometry confirmed the efficient inhibition of cytotoxic T cells. As revealed, the inhibitor effectiveness could attain more than 90% in both spleen and tumor tissues (Figure 9e). In conclusion, Bio-MnO_2_ NPs plus radiation could activate immune responses in vivo, and cytotoxic T cells performed a major role.

## 4. Conclusions

Herein we proposed an easily accessible multifunctional nanoplatform Bio-MnO_2_ NPs and verified its unique features involving highly efficient capability of generation of oxygen and consumption of overproduced H_2_O_2_ so as to reconstruct a tumor normoxic microenvironment. MnO_2_ NPs and RT play synergistic roles in ROS-induced tumor ablation. However, their combined effect in anti-tumor immunity and activation of specific immune pathways have not been proposed as of now. Our research indicated that Bio-MnO_2_ NPs plus RT activated strong immune responses via the cGAS/STING pathway and ICD, further supporting infiltration of immune cells especially CTLs, killing tumor cells while converting immunosuppressive microenvironment. This strategy showed great potential to circumvent multiple clinical challenges of RT with a single reagent and offered the possibility of combining nanomaterials plus RT with immunotherapy.

For clinical translation of this combined therapy, it would be valuable to explore the immune landscape in more detail in the future, including the tumor-associated macrophages (TAMs) polarization, memory T cells induction and regulatory T cells (Tregs) suppression, long-term inhibition of tumor re-growth and metastasis. These studies may provide more new insights into the immune activating mechanisms of this combined therapy. Moreover, it is worth noting that a key approach to improve immunotherapy has been to combine RT with checkpoint inhibitors including cytotoxic T lymphocyte-associated antigen 4 (CTLA-4) and the PD-1/PD-L1 to induce systemic immune responses [66,67]. Given our gratifying achievements in Bio-MnO_2_ NPs plus RT, we could anticipate that the triple therapy involving Bio-MnO_2_ NPs, RT and checkpoint inhibitors may manifest more surprising effects. Recent research found that manganese oxide nanomaterials as an oxygen supplier could reduce PD-L1 expression by overcoming tumor hypoxia [68], which also provided a possible mechanism and notion for the triple treatment with PD-1/PD-L1 inhibitor.

## Data Availability

Not applicable.

## Supplementary Materials

The following supporting information can be downloaded at: https://www.mdpi.com/article/10.3390/nano12183138/s1; Table S1: Primer sequences used for amplification; Table S2: Antibodies used in this research; Figure S1: Uptake of Cy3 labelled Bio-MnO_2_ NPs at different time intervals; Figure S2: Bio-MnO_2_ NPs enhanced ROS production in H520 cells; Figure S3: H&E staining in various major organs (heart, liver, spleen, lung and kidney); Figure S4: Flow cytometry of CD45^+^CD3^+^ T cells and CD45^+^CD3^+^CD8^+^ T cells in spleens; Figure S5: Tumor growth curve of each mouse after different treatments.
