# Supplementary material for: Biomineralized Manganese Oxide Nanoparticles Synergistically Relieve Tumor Hypoxia and Activate Immune Response with Radiotherapy in Non-Small Cell Lung Cancer
Source: Nanomaterials (Basel). 2022 Sep 10;12(18):3138. doi: 10.3390/nano12183138 (PMC9501587; doi:10.3390/nano12183138)
Supplement: Supplementary file 1 [file nanomaterials-12-03138-s001.zip › nanomaterials-1858093-supplementary.pdf]

Supplementary files of

Biomineralized Manganese Oxide Nanoparticles Synergistically Relieve Tumor Hypoxia and Activate Immune Response with Radiotherapy in Non-Small Cell Lung Cancer

Xinyu Liu [1,†], Meron Tsegay Kifle [2,†], Hongxin Xie [1,3,†], Liexi Xu [1], Maoling Luo [1], Yangyi Li [1], Zhengrong Huang [1,3], Yan Gong [3,4,*], Yuzhou Wu [2,*] and Conghua Xie [1,5,6,7,*]

1. Department of Radiation and Medical Oncology, Zhongnan Hospital of Wuhan University, Wuhan, China
2. Hubei Key Laboratory of Bioinorganic Chemistry and Materia Medica, Hubei Engineering Research Center for Biomaterials and Medical Protective Materials, School of Chemistry and Chemical Engineering, Huazhong University of Science and Technology, Wuhan, China
3. Department of Biological Repositories, Zhongnan Hospital of Wuhan University, Wuhan, China
4. Tumor Precision Diagnosis and Treatment Technology and Translational Medicine, Hubei Engineering Research Center, Zhongnan Hospital of Wuhan University, Wuhan, China
5. Hubei Key Laboratory of Tumor Biological Behaviors, Zhongnan Hospital of Wuhan University, Wuhan, China
6. Hubei Cancer Clinical Study Center, Zhongnan Hospital of Wuhan University, Wuhan, China
7. Wuhan Research Center for Infectious Diseases and Cancer, Chinese Academy of Medical Sciences, Wuhan, China

[*] Correspondence: yan.gong@whu.edu.cn (Y.G.); wuyuzhou@hust.edu.cn (Y.W.); chxie_65@whu.edu.cn (C.X.)

† Theses authors contributed equally to this work.

Tables

Table S1. Primer sequences used for amplification.

**Gene**	**Sequences or target sequence (5'→ 3')**
GAPDH-FP	CTGTTCGACAGTCAGCCGCATC
GAPDH-RP	GCGCCCAATACGACCAAATCCG
CCL5-FP	TGCCCACATCAAGGAGTATTT
CCL5-RP	CTTTCGGGTGACAAAGACG
CXCL10-FP	GGCCATCAAGAATTTACTGAAAGCA
CXCL10-RP	TCTGTGTGGTCCATCCTTGGAA
IFNβ-FP	AACTTGCTTGGATTCCTACAAAG
IFNβ-RP	TATTCAAGCCTCCCATTCAATTG

Table S2. Antibodies used in this research.

**Antibody**	**Company**	**Catalog number**
Ki-67	Proteintech	27309-1-AP
BCL-2	Proteintech	60178-1-Ig
BAX	Proteintech	60267-1-Ig
P-γH2AX	ABclonal	AP0687
BRCA1	Proteintech	22362-1-AP
PARP1	Proteintech	66520-1-Ig
KU80	Proteintech	16389-1-AP
KU70	Proteintech	10723-1-AP
RAD51	Proteintech	14961-1-AP
dsDNA	Abcam	ab27156
P-IRF3	Cell Signaling	37829
IRF3	Proteintech	11312-1-AP
P-TBK1	Cell Signaling	5483
TBK1	Cell Signaling	3504
P-STING	Cell Signaling	50907
STING	Proteintech	19851-1-AP
GAPDH	Proteintech	10494-1-AP
Ms CD45 APC-Cy7	BD Pharmingen	557659
Ms CD3e FITC	BD Pharmingen	553061
Ms CD4 APC	BD Pharmingen	553051
Ms CD8a PE	BD Pharmingen	553032

Figures

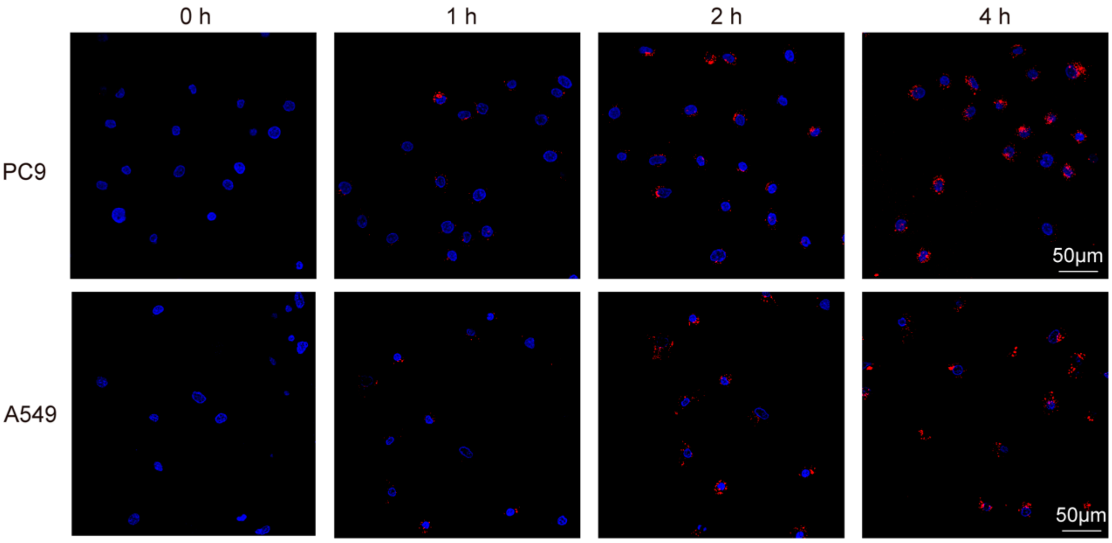

Figure S1. Uptake of Cy3 labelled Bio-MnO_2 NPs at different time intervals (0 h, 1 h, 2 h, 4 h). Scale bar, 50 μm.

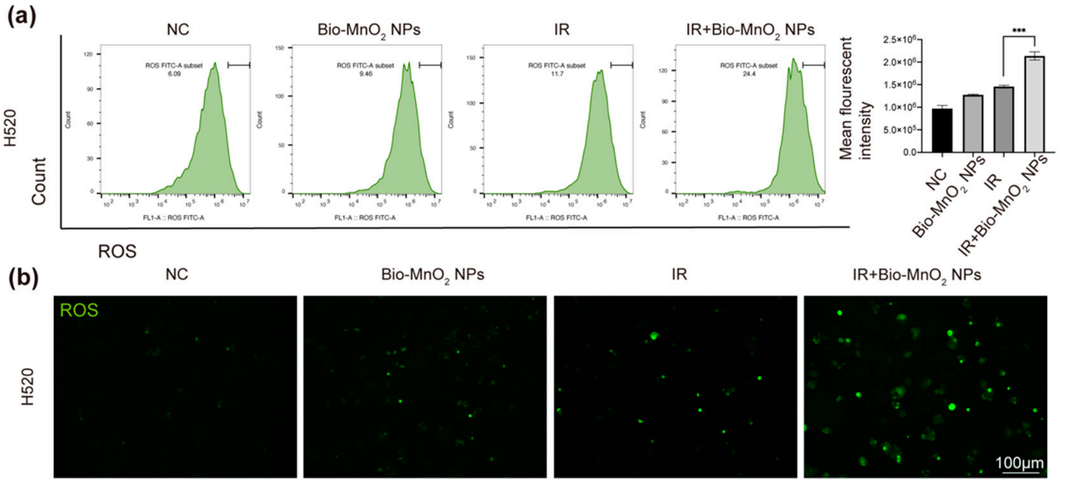

Figure S2. Bio-MnO_2 NPs enhanced ROS production in H520 cells. (A) Flow cytometry of ROS in H520 cells with different treatments. (B) Representative DCF staining images of H520 cells. Scale bar, 100 μm. ***, $p < 0.001$.

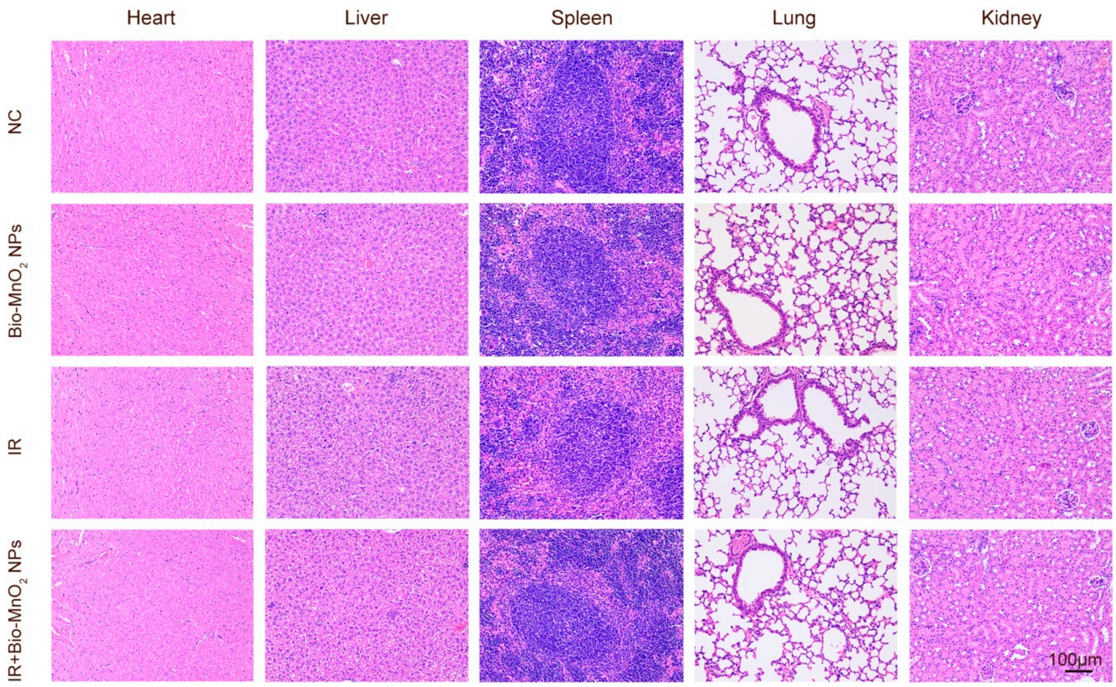

Figure S3. H&E staining in various major organs (heart, liver, spleen, lung, and kidney). Scale bar, 100 μm.

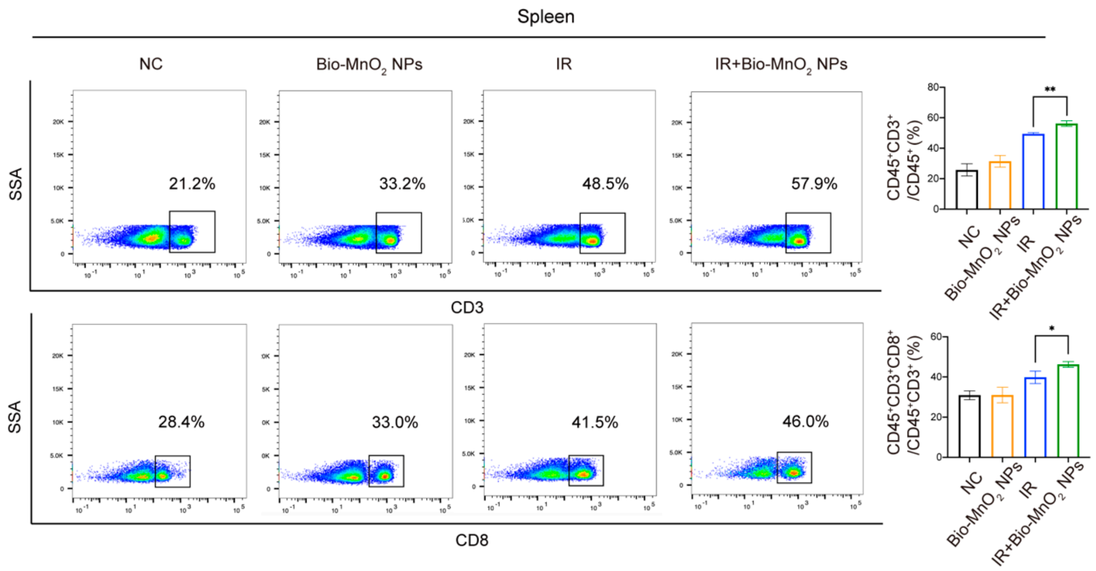

Figure S4. Flow cytometry of $CD45^+CD3^+$ T cells and $CD45^+CD3^+CD8^+$ T cells in spleens. *, $p < 0.05$; **, $p < 0.01$.

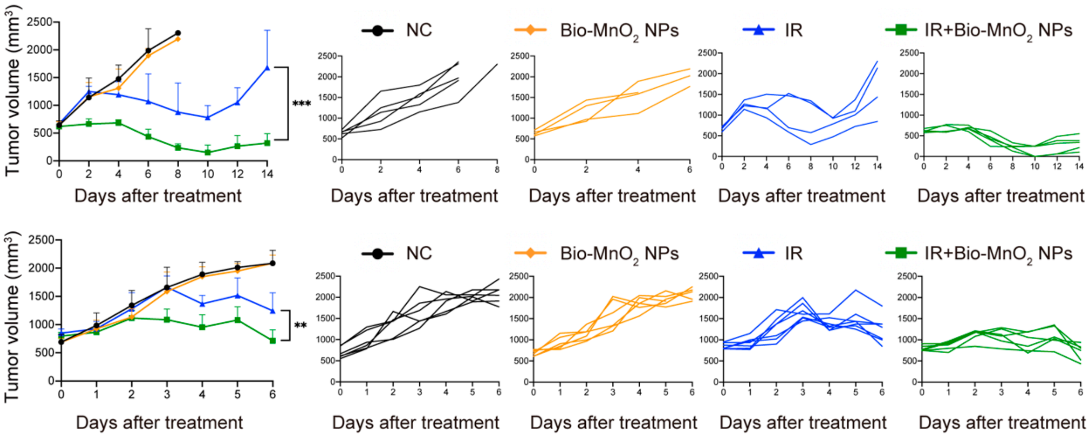

Figure S5. Tumor growth curve of each mouse after different treatments: negative control (NC), Bio-MnO_2 NPs, IR, and IR + Bio-MnO2 NPs. **, $p < 0.01$. ***, $p < 0.001$.
